# Immunosuppressive Treatments and Risk Factors Associated with Non-Response to Hepatitis B Vaccination: A Cohort Study

**DOI:** 10.3390/vaccines13020184

**Published:** 2025-02-14

**Authors:** Raquel Padilla-Matas, Victoria Salguero-Cano, Eva Soler-Iborte, Javier Baca-Hidalgo, Marta Pérez-Dionisio, Soledad Gutiérrez-Linares, Inmaculada Guerrero-Fernández de Alba, María del Carmen Valero-Ubierna, María Fernández-Prada, Mario Rivera-Izquierdo

**Affiliations:** 1 Service of Preventive Medicine and Public Health, Hospital Universitario San Cecilio, 18016 Granada, Spain; raquel.padilla.sspa@juntadeandalucia.es (R.P.-M.); eva.soler.sspa@juntadeandalucia.es (E.S.-I.); javier.baca.sspa@juntadeandalucia.es (J.B.-H.); marta.perez.dionisio.sspa@juntadeandalucia.es (M.P.-D.); soledad.gutierrez.linares.sspa@juntadeandalucia.es (S.G.-L.); inmaculada.guerrero.f.sspa@juntadeandalucia.es (I.G.-F.d.A.); mariac.valero.sspa@juntadeandalucia.es (M.d.C.V.-U.); 2 Doctorate Program in Clinical Medicine and Public Health, University of Granada, 18012 Granada, Spain; 3 Instituto de Investigación Biosanitaria de Granada, ibs.GRANADA, 18012 Granada, Spain; mariorivera@ugr.es; 4 Preventive Medicine Service, Alvarez Buylla Hospital of Mieres, Mieres, 33611 Asturias, Spain; maria.fernandezp@sespa.es; 5 Department of Preventive Medicine and Public Health, University of Granada, 18016 Granada, Spain; 6 CIBER de Epidemiología y Salud Pública (CIBERESP), 28029 Madrid, Spain

**Keywords:** monoclonal antibodies, biologics, elderly, smoking, Andalusia

## Abstract

**Background**: The aim of this study was to evaluate the serological response after the complete hepatitis B vaccination of patients according to the immunosuppressive treatment they underwent, and to identify potential factors associated with non-responders. **Methods**: A prospective cohort study was conducted, and patients under immunosuppressive therapies were considered exposed. The main outcome was non-response to hepatitis B vaccination. Bivariate analysis was conducted to detect differences between exposed and non-exposed patients. A multivariable log-binomial regression model was designed to analyze potential factors independently associated with non-responders. **Results**: A total of 289 patients were included. Immunosuppressive treatment was associated with non-response to hepatitis B vaccination (RR = 2.49, 95% CI: 1.26–4.96). Concretely, the use of cytotoxic therapies showed increased risk, although anti-CD20 and anti-JAK also showed a tendency to be associated with non-responders. Other variables associated with non-responders were older age (6–7% higher risk per year), smoking (RR = 3.08, 95% CI: 1.41–6.74) and certain vaccine regimens. These findings were similar for persistent non-responders despite an additional booster dose. **Conclusions**: Patients receiving immunosuppressive treatments, who are older in age or who are smokers have a higher risk of non-response to conventional hepatitis B vaccination. These data might serve to optimize hepatitis B vaccination in high-risk patients.

## 1. Introduction

Immunosuppressed patients have a limited response to the production of antibodies against the hepatitis B surface antigen (HBsAg). For example, only 61% of patients with inflammatory bowel disease, either because of their autoimmune disease or because of concomitant immunosuppressive treatments, respond adequately to vaccination against hepatitis B virus (HBV) [1]. Similarly, a poor serological response, as well as an alarming absence of specific recommendations, has also been described in patients infected with human immunodeficiency virus [2]. This has led the main international guidelines of specialties related to immunosuppressive diseases (e.g., rheumatology) to recommend specific HBV vaccination schedules for these patients [3]. HBV vaccination coverage with different vaccines has been increased worldwide, which has led to a substantial reduction in hepatitis B-related morbidity and mortality [4,5]. In Spain, pediatric patients receive vaccination in primary care settings, whereas at-risk adults (e.g., immunocompromised) receive vaccination in specialized consultations from preventive medicine and public health services. In Andalusia (a region of Southern Spain with 8.6 million inhabitants), information and instructions for the vaccination of at-risk groups are available from the Ministry of Health and Consumer Affairs (Andalusian Regional Government) [6]. Table A1 (Appendix A) shows the monovalent hepatitis B vaccines available in Andalusia.

Currently, there are a multitude of immunosuppressive treatments (including monoclonal antibodies) that are administered for the treatment of various chronic and acute pathologies. Patients with these treatments receive HBV vaccination following a protocol and according to the results of a previous serology. However, the response to this vaccination has not been sufficiently studied. Our research group published an exhaustive analysis of all commercialized monoclonal antibodies, as well as updated vaccination recommendations according to the level of immunosuppression, up until 2020 [7], that have recently been updated to 2024 [8,9]. To date, these are the only articles published detailing the overwhelming number of immunosuppressive monoclonal antibodies used in clinical practice, as well as their mechanisms of action, indications, immunosuppressive capacity, and need for specific vaccination based on the data in the technical data sheet.

However, for a significant number of patients receiving these treatments, there is frequently an insufficient level (quantification) of hepatitis B surface antibodies (antiHBs) after vaccination. This could imply incorrect immunization against HBV, even after the administration of booster doses of vaccine after their primary vaccination. This raises the need to identify which immunosuppressive treatments affect the vaccine response, since these data could justify a more effective individualized vaccination strategy to generate protective immunity. Nevertheless, there is a lack of studies analyzing specifically the role of immunosuppressive therapies on the HBV vaccine response. This data would serve to propose individualized vaccination strategies according to the specific treatment or disease of each patient. This would optimize the current homogeneous vaccination strategies proposed in clinicals protocols (mostly based on expert recommendations and not evaluated), which do not distinguish between responders and non-responders.

The aim of this study was to evaluate the serological antiHBs response after the complete vaccination of patients according to immunosuppressive treatments, and to identify potential factors associated with non-responders.

## 2. Materials and Methods

The STROBE (strengthening the results of observational studies in epidemiology) guidelines were followed to report the results of this observational study [10].

### 2.1. Study Design and Setting

The study design was that of a prospective cohort (observational, longitudinal) study. All patients were consecutively selected from the vaccination unit of the Service of Preventive Medicine and Public Health, Hospital Universitario San Cecilio, Granada (Spain). All patients included in the sample had an indication for a complete HBV vaccination schedule according to their disease or treatment. All patients included had baseline negative antiHBs serology prior to vaccination. The recruitment period started on 1 September 2022 and continued to 1 July 2023, the end of the follow-up. Patients that did not want to voluntarily participate in the study or that were unable to understand the objectives of the study (i.e., previous diagnosis of dementia or mental health disorders) were excluded. Exposed participants were patients under immunosuppressive treatments that received vaccination against HBV. Non-exposed participants were patients without immunosuppressive treatments that received vaccination against HBV for other reasons. The sample size was calculated a priori. Assuming that around 50% of patients attended to in our vaccine unit had immunosuppressive treatments (according to a pilot analysis), a total of 280 patients (140 exposed and 140 non-exposed) were needed to reach an alpha error of 5% and beta error of 20%. To reach that figure, assuming a small percentage of losses to follow-up, a total of 300 participants included in the study were estimated.

In our vaccine consultation, a homogeneous vaccination schedule was recommended for all patients according to our regional protocols [6]. This schedule consisted of doses of monovalent HBV vaccine (Engerix 20 µg/mL of HBsAg or HBVaxpro 10 µg/mL of HBsAg) at 0, 1 and 6 months (at first visit, at 1 month from the first visit and at 6 months from the first visit) except for patients in a pre-dialysis or dialysis program with chronic renal failure, that received doses of Fendrix (20 µg/mL of HBsAg adjuvanted AS04C 50 µg, 0.5 mL) at 0, 1, 2 and 6 months. This was followed by a serologic test 2 months after the last dose, resulting in a total of 8 months of follow-up for all participants. Therefore, all participants received a serologic test 8 months from the first vaccine dose, according to our clinical protocols.

Patients showing a negative result after the conventional schedule received another booster dose 8 months after the first visit. Two months after, another serologic test is indicated. Therefore, non-responders had a total of 10 months of follow-up.

### 2.2. Variables of the Study

The exposure variable was the presence of an immunosuppressive treatment at the time of receiving the first dose of the vaccine against HBV. We also decided to explore the specific effect of monoclonal antibodies (MABs) according to previous research [9].

The main outcome variable was the serological quantification of hepatitis B surface antibodies (antiHBs, mU/mL) 8 months after the beginning of the vaccination regimen (at 0, 1 and 6 months), which is 2 months after completion of the schedule. The results of the quantification included values between 10 mU/mL (minimum detectable number) and 1000 mU/mL (maximum detectable number) according to cut-off points of the microbiology laboratory of our hospital. This variable was subsequently dichotomized as positive (≥10 mU/mL) or negative (<10 mU/mL; no detection). The presence of negative quantification after a complete regimen was considered as non-response.

These patients received booster doses in addition to the conventional regimen per protocol. If, despite the booster dose, they remained negative in the antibody titration, they were considered as persistent non-responders (secondary outcome). So, there were two outcome variables: no antibody response after conventional regimen (non-responders) and no antibody response after booster doses (persistent non-responders). The rest of the variables that were collected for the study were as follows:

(1) Type of monovalent HBV vaccination received. The monovalent vaccines available in our study were Engerix (20 µg/mL of HBsAgs) and HBVaxpro 10 (10 µg/mL of HBsAg), used for adults 16 years and up for conventional vaccination schedules, and Fendrix (20 µg/mL of HbsAg adjuvated AS04C 50 µg, 0.5 mL) or HBVaxpro 40 (40 µg/mL of HBsAg), used for patients in dialysis or pre-dialysis programs with chronic renal failure. According to our protocols, and given that no HBVaxpro 40 was used in our sample, we divided de type of vaccine as follows: only-Engerix regimen, only-HBVaxpro 10 regimen, a combination of both vaccines according to availability, or only-Fendrix regimen; (2) the need for booster doses (yes/no); (3) the vaccination dates; (4) the type of immunosuppressive treatment received; (5) the immunosuppressant family according to its therapeutic target; (6) sociodemographic variables (age, sex and smoking status) and (7) clinical variables: main diagnosis (the primary diagnosis that led the patient to receive immunosuppressive therapy or vaccination), previous diagnoses, concurrent treatments received, history of prior HBV vaccination and history of previous viral hepatitis infections diagnosed in the clinical records.

### 2.3. Data Sources

Patients who attended the vaccine consultation were recorded in the databases of the Preventive Medicine and Public Health service, along with information on the date and the type of vaccination received. Clinical and laboratory data from the medical records and microbiology laboratory results were consulted. The database was anonymized and expanded in a spreadsheet, including the study variables previously detailed, for the purposes of the study.

### 2.4. Statistical Analyses

First, descriptive univariate analysis was performed to characterize the sample. Means and standard deviations were calculated for quantitative variables, and absolute frequencies and percentages were calculated for qualitative variables, stratified by exposure groups (immunocompromised vs. non-immunocompromised).

Second, the incidence of “non-responders” and “persistent non-responders” was calculated and stratified by the exposure (immunosuppressive treatment). Risk ratios and their corresponding 95% confidence intervals (95% CIs) were calculated. A *t*-test was performed to analyze differences in serological quantification according to exposure.

Third, multivariable analyses were conducted to quantify the effect of immunosuppressive treatments on the antiHBs serological response, adjusting for the covariates collected. Log-binomial (generalized linear) regression models were adjusted for the outcomes “non-responders” and “persistent non-responders” to obtain adjusted relative risks. All analyses were performed using Stata (StataCorp^®^, College Station, TX, USA), version 15.0.

### 2.5. Ethical Considerations

The requirements established by the Declaration of Helsinki for research with human data were met. An anonymized database with no potentially identifiable variables was used. The protocol of the study was approved by the Provincial Ethical Research Committee, Granada, code 1675-N-23.

## 3. Results

### 3.1. Description of the Sample

**Figure 1:**
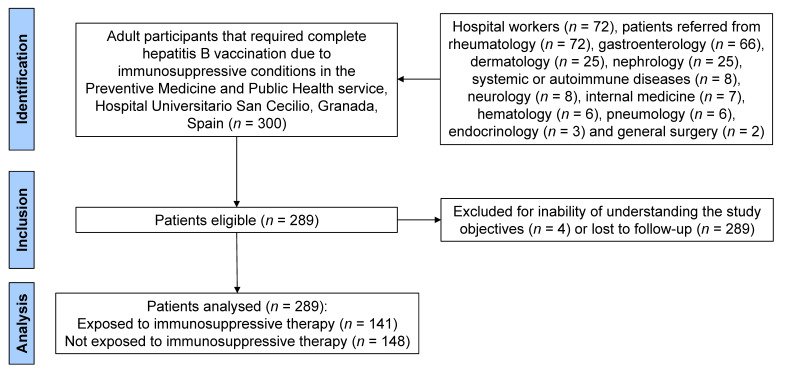
Flow chart of the study sample selection.

The sample of the cohort study involved a total of 289 patients (Figure 1). Of them, 141 (48.8%) were considered exposed (receiving immunosuppressive treatment at the time of the first vaccination consultation) and 148 (51.2%) were considered non-exposed (not receiving immunosuppressive treatment). All participants in the study had an indication for vaccination at the Preventive Medicine Service of the San Cecilio Hospital. A total of 72 (24.9%) participants were hospital workers, and 217 (75.1%) were patients coming from a hospital service that requested vaccination according to a pathology, treatment, or expected immunosuppression in the future. The most frequent referral services were rheumatology (*n* = 72, 24.9%), digestive/gastroenterology (*n* = 66, 22.8%), dermatology (*n* = 25, 8.7%), nephrology (*n* = 25, 8.7%) and systemic or autoimmune diseases (*n* = 8, 2.8%). The most frequent digestive disorders were ulcerative colitis (*n* = 33, 11.4%) and Crohn’s disease (*n* = 27, 9.3%), the most frequent dermatological disorders were psoriasis (*n* = 32, 11.1%) and Sjögren syndrome (*n* = 3, 1.0%), and the most frequent systemic disease was multiple sclerosis (*n* = 7, 2.4%).

The main characteristics of the sample, stratified by exposure, are shown in Table 1. A total of 173 (59.9%) participants were women, and the mean age of the sample was 47.9 years (standard deviation: 14.6). There were no baseline differences according to sex, age or smoking status in the comparison groups (exposed and non-exposed). Table 1 shows the main diagnoses of the comparison groups.

The exposed group (immunosuppressive treatment) had a higher frequency of autoimmune disease, arthritis or spondylitis and dermatological disorders. The non-exposed group (without an immunosuppressive treatment) was composed of a higher number of patients with chronic kidney disease or dialysis. Digestive diseases, mostly of the inflammatory bowel disease type, were similarly distributed in both groups.

Regarding the exposed group, there was a wide variety of immunosuppressive therapies. Table 2 summarizes the main immunosuppressive treatments received. Briefly, 97 (68.8%) patients received immunosuppressive treatments with MABs, commonly included in the category of biological drugs, 72 (51.1%) received non-MAB chemotherapeutic or cytotoxic treatments, and 7 (5.0%) received other immunosuppressive treatments. The most frequent specific immunosuppressive drugs in the sample were adalimumab (*n* = 58, 41.1% of the exposed), methotrexate (*n* = 43, 30.5% of the exposed) and azathioprine (*n* = 14, 10.0% of the exposed).

### 3.2. Incidence and Factors Associated with the Outcomes (Non-Responders to Hepatitis B Vaccination and Persistent Non-Responders After a Booster Dose)

A total of 75 patients (26.0%) were non-responders to the conventional vaccination schedule, and 28 (9.7%) were non-responders despite an additional booster dose (persistent non-responders). The cumulative incidences of each variable for both outcomes were calculated. Table 3 shows these results, as well as the crude risk ratio (cRR) by group.

Of the 213 patients with serological response, the exposed (immunosuppressed) group showed a mean quantification of 535.0 mU/mL (sd = 45.1) and the non-exposed (not immunosuppressed) group showed a mean quantification of 764.9 mU/mL (sd = 82.4) (*p*-value of *t*-test = 0.0145).

The exposure (receiving immunosuppressive treatment) was associated with non-response to the conventional vaccination schedule. Only six patients received multiple immunosuppressive drugs, and all of them were non-responders. In addition, the main sociodemographic factors associated with this outcome were being aged over 55 years and smoking status (being an active or ex-smoker). Only arthritic or spondylosis conditions were associated with non-response to vaccination. The treatments associated with non-response were monoclonal antibodies, especially infliximab (anti-TNF), tocilizumab (anti-IL-6) and anti-CD20 (rituximab and ocrelizumab). Similarly, non-MAB chemotherapeutics also showed a strong association with an absence of HBV vaccine response, especially methotrexate and mycophenolate. Finally, both anti-JAK and other immunosuppressants (fingolimod and leflunomide) also showed an association. The complete Vaxpro or combined (Vaxpro and Engerix) vaccination regimen was more favorable than the single Engerix regimen. Nevertheless, the vaccine regimens depended on the status of the exposure (immunosuppression), as shown in Table A2 (Appendix A). Briefly, patients receiving Fendrix were patients under dialysis or pre-dialysis regimens with a mean age of 61.1 years old (sd = 3.2), compared to a mean age of 46.6 years old (sd = 0.9) in patients receiving other vaccines, and patients receiving Engerix showed a higher frequency of immunosuppression treatments.

Regarding the secondary outcome, non-response to vaccination despite receiving another booster dose of vaccine (persistent non-responders), associations were identified in smokers and those diagnosed with arthritis or spondylosis and systemic diseases, as well as patients being treated with anti-CD20 (especially infliximab), non-MABs cytotoxic drugs (especially methotrexate) or anti-JAKs. Notably, all patients receiving anti-CD20 (*n* = 5) or anti-JAK (*n* = 3) treatments who did not respond to the conventional regimen also failed to respond following booster doses. The associations for type and schedule of vaccination were the same as for the main outcome.

### 3.3. Adjusted Results from the Multivariable Regression Models

**Figure 2:**
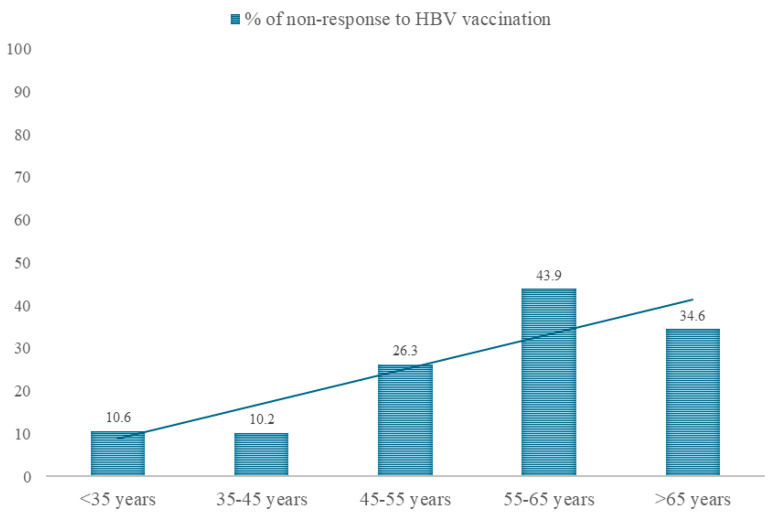
Percentage of non-response to hepatitis B vaccination (HBV) according to age group.

First, multivariable log-binomial regression models were performed using non-response to the conventional vaccination schedule as the dependent (explained) variable. As independent (explanatory) variables, the main risk factors observed in the previous analysis (unadjusted RRs) were explored. Given that age showed an approximately linear relationship with non-response to vaccination (Figure 2), it was included as a quantitative variable in the adjusted models.

Table 4 summarizes the main results obtained when adjusting for different variables. Analyses show that the variables that were consistently associated with non-response in all settings were immunosuppressive treatments (2.5 times higher risk), older age (6–7% higher risk per year of age) and an Engerix-only regimen (5–6 times higher risk than all other regimens). Cytotoxic drugs or non-MAB chemotherapy drugs were also very consistently associated with non-response to vaccination.

Given the small number of outcomes for persistent non-response despite booster doses (*n* = 24), detailed multivariable analysis results are not shown. In any case, the findings were very similar; adjusting for age, sex, baseline diagnosis, immunosuppression and vaccination, the only variables consistently associated with persistent non-response were age (OR = 1.05, 95% CI: 1.02–1.09), exclusive vaccination schedule with Engerix (OR = 2.95, 95% CI: 1.00–8.93) and active or past smoking status (OR = 5.40, 95% CI: 1.40–20.85).

## 4. Discussion

We present the results of a study with a cohort of patients and hospital workers who received vaccinations against HBV (exposed and unexposed to immunosuppressive treatment) and their serological responses (antiHBs quantification) 8 months after the vaccination schedule. Our data showed that over a third of patients under immunosuppressive treatments were non-responders (36.9%), which contrasts with data in the general population (5% to 20%) [11,12], and 14.2% were persistent non-responders after a booster dose. After adjusting for the main potential confounders, we found that the variables associated with non-response to vaccination were age (6–7% higher risk per year), the exclusive Engerix regimen (5–6 times more than the other regimens), immunosuppressive treatments and the use of cytotoxic or non-MABs chemotherapy drugs. For persistent non-responders, despite booster doses, the findings were similar: when adjusted, the only variables associated with serological non-response were age, an exclusively Engerix regimen and smoking status (ex-smoker or active smoker).

The influence of age on the immune response to vaccines is a well-known factor. The constant response in patients aged <45 years in our sample can be explained by the vaccination calendar (they received vaccination doses during their childhood), and the relative higher risk in patients aged 55–65 compared with those over 65 years can be explained by the sample selection (most patients in dialysis receiving AS04C-adjuvanted vaccines are in this subgroup). As AS04C-adjuvanted vaccines have proven to be more immunogenic, this may explain the relatively lower percentage of non-responders in this subgroup compared to patients aged 55–65 years old. In any case, there is a clear increase in non-responders in patients aged over 55 years. In older people, immunosenescence decreases the ability of the immune system to respond effectively to infections, as well as to vaccines [13]. A meta-analysis found a reduced response to hepatitis B vaccination in adults aged 40 years and older [14]. Asan et al. [15], in a study conducted on patients receiving dialysis, found an increase in non-response of 2.6 times in those over 65 years compared to younger people. Similarly, Vermeiren et al. [16] found in their cohort study that immunosenescence already appears at an early age. All these data are consistent with the findings of our study, which showed a decrease in response to vaccination of 6–7% per year of age.

Receiving an immunosuppressive treatment (our exposure variable) was also associated with non-response to HBV vaccination, which is consistent with a meta-analysis published by Qiu et al. [17]. Hepatitis B vaccines are safe and highly recommended for immunocompromised patients [18], although higher than usual doses may be necessary to obtain an optimal response [19]. This could explain why in our study the Engerix-only regimen was associated with a lower response than the other regimens, as its composition is lower (20 µg/1 mL) than that of the other vaccines analyzed [20]. We found no randomized clinical trials comparing monovalent HBV vaccines in immunocompromised patients. However, for patients aged over 40 years, a clinical trial conducted in Belgium showed 71% seroprotection with Vaxpro, 80% with Engerix and 92% with Twinrix (a combination hepatitis A and B vaccine) [21]. It is possible that patients undergoing immunosuppressive treatments could require improved vaccines, such as the AS04C-adjuvanted Fendrix vaccine, as occurs to patients under dialysis. As well, two newer HBV vaccines have been licensed in the European Union: Heplisav B (a CpG-adjuvanted vaccine) and PreHevbrio (a three-antigen HBV vaccine). Future studies should analyze their potential benefits for immunocompromised patients.

Additionally, patients receiving each vaccine presented different with conditions and immunosuppression statuses, as presented in Table A2. The combined Engerix and Vaxpro regimen was, unfortunately, at random (depending on the availability of vaccines at each time). Therefore, this was a very heterogeneous group regarding vaccination regimens (some patients started with one vaccine and finished with another, and some patients received two doses of Engerix whilst others received two doses of Vaxpro). In any case, this study was not designed to analyze differences between vaccines, therefore these results must be considered with caution, and future studies should be conducted to test the best vaccine and schedule for immunocompromised patients.

Among the different treatments used, the monoclonal antibodies infliximab and tocilizumab as well as rituximab and ocrelizumab were associated with worse vaccine responses, with no response despite the booster dose, as with anti-JAK. One possible explanation may lie in immunological mechanisms related to the immune response to vaccines. Hepatitis B antibody formation requires the optimal induction of follicular T cells as well as memory B cells [22], processes that are altered to a greater or lesser extent in the administration of these treatments.

Another variable associated with non-response to vaccination in our study was being an active or ex-smoker. In their review [23], Zimmerman and Curtis found numerous studies with the same result but a few others with no statistical differences. A cohort study with a 24 year follow-up [24] found an association between being a smoker and having hepatitis B antibodies below the safety threshold when compared to the non-smoking population. Tobacco smoke contains around 7000 different chemicals [25], and there is growing evidence that smoking and its constituents significantly influence the regulation of immunity. This might be mediated by molecular mechanisms, including increased levels of pro-inflammatory cytokines, such as tumor necrosis factor-α (TNF-α), interleukin (IL)-1, IL-6 and IL-8, and decreased levels of anti-inflammatory cytokines, such as IL-10 [26].

This study has some limitations. First, it is a single-center study. However, we included both patients and center workers, and the baseline characteristics of both groups were similar. Nevertheless, the external validity of the results may be limited by the type of population and the country (Spain). Second, the main outcome variable (non-response to vaccination) could be influenced by the type of vaccine rather than by immunosuppression, as some immunosuppressed patients were often vaccinated with Engerix, which may be less effective than other regimens; also, collecting data on previous HBV exposure through anti-HBc serology would have been of value, but was not included, as it was not systematically solicited in our vaccine consultation, according to regional clinical protocols. To approach this issue, we conducted multivariable analyses, including the type of vaccine as a covariate. Third, we included a very heterogeneous population, as the degree and type of immunosuppression may differ considerably among diagnoses and treatments. We aimed to include a representative population for the variety of patients in the adult vaccine consultation. To deal with this issue, we included the diagnosis and treatment as covariates in the adjusted models. Fourth, some relevant variables, like smoking status, showed missing data in a substantial number of patients. As we do not know the reasons for this missing data (missing at random or due to unknown biases), the analyses conducted with these variables should be cautiously considered. We did not analyze how long the production of antibodies lasts, as we established a common period for the serologic test (8 months after the first dose); future studies should analyze the production period in the long term. Finally, the number of patients with some treatments, such as anti-JAK, was too low to draw robust conclusions. Similarly, we could not design multivariable models for the secondary outcome (persistent non-response after a booster dose) given the limited number of participants that developed such an outcome. To minimize potential biased associations, multivariate analyses were conducted, adjusting for the main confounders considered in the study. Future multicenter studies including larger sample sizes should corroborate our results.

## 5. Conclusions

Patients receiving immunosuppressive treatments have a lower serological response to hepatitis B vaccination than those not receiving immunosuppressive treatments. Potential factors related to non-responders, after adjusting for our main confounding variables, were age (6–7% lower response for each year), smoking status (former or current smoker) and certain types of treatments. Our data underline the importance of choosing the right vaccination schedule for at-risk patients, as well as recommending smoking cessation to improve a patient’s immune system. Moreover, vaccines with higher doses showed lower non-response rates. Our findings suggest that immunocompromised patients should receive improved vaccines (i.e., high-dose or AS04C-adjuvanted vaccines) against HBV. Future studies should specifically analyze the response of specific immunosuppressive therapies to vaccination. Our data may serve to optimize hepatitis B vaccination schedules for patients undergoing immunosuppressive treatments.

## Figures and Tables

**Table 1:** Sociodemographic characteristics and clinical diagnoses, stratified by exposure to immunosuppressive (IS) treatments.

Variable	Total (*n* = 289)	Exposed (IS Treatment) (*n* = 141)	Non-Exposed (no IS Treatment) (*n* = 148)
	*n* (%)/x (s)	*n* (%)/x (s)	*n* (%)/x (s)
Sex, *n* (%)			
Women	173 (59.9)	87 (61.1)	86 (58.1)
Men	116 (40.1)	54 (38.3)	62 (41.9)
Age, x (SD)	47.9 (14.6)	48.6 (14.3)	47.1 (15.0)
Age, *n* (%)			
<35 years	47 (16.3)	21 (14.9)	26 (17.6)
35–45 years	49 (17.0)	26 (18.4)	23 (15.5)
45–55 years	80 (27.7)	41 (29.1)	39 (26.4)
55–65 years	58 (20.1)	37 (26.2)	21 (14.2)
>65 years	55 (19.0)	16 (11.4)	39 (26.4)
Smoking status, *n* (%)			
Current smoker	49 (27.4)	27 (24.8)	22 (31.4)
Ex-smoker	44 (24.6)	26 (23.9)	18 (25.7)
Non-smoker	86 (48.0)	56 (51.4)	30 (42.9)
Unknown	110 (38.1)	-	-
Main diagnosis ^1^			
Arthritis or spondylitis	69 (23.9)	63 (44.7)	6 (4.1)
Digestive disorders	62 (21.5)	27 (19.2)	35 (23.7)
Dermatological disorders	48 (16.6)	45 (31.9)	3 (2.0)
CKD-dialysis	23 (8.0)	0 (0.0)	23 (15.5)
Systemic disease	19 (6.6)	14 (9.9)	5 (3.4)
Transplant	4 (1.14)	1 (0.7)	3 (2.0)
Neoplasia	3 (1.0)	0 (0.0)	3 (2.1)
Splenectomy	1 (0.4)	1 (0.7)	0 (0.0)

CKD: chronic kidney disease. ^1^ The overall percentage is higher than 100% because there are patients with more than one diagnosis, or with diagnoses belonging to two categories (e.g., psoriatic arthritis).

**Table 2:** Types of immunosuppressive treatments received by the exposed group (*n* = 141).

Type of Immunosuppressive Treatment	*n* (%) ^1^
Monoclonal antibodies (MABs)	97 (68.8)
Anti-TNF	70 (49.6)
Adalimumab	58 (41.1)
Infliximab	9 (6.4)
Etanercept	5 (3.5)
Certolizumab-pegol	1 (0.7)
Anti-IL-6 (tocilizumab)	6 (4.3)
Anti-CD-20	5 (3.5)
Rituximab	3 (2.1)
Ocrelizumab	2 (1.4)
Anti-IL-12/23 (ustekinumab)	2 (1.4)
Anti-IL-13 (tralokinumab)	1 (0.7)
Anti-IL-17	7 (5.0)
Secukinumab	6 (4.3)
Bimekizumab	1 (0.7)
Ixekizumab	1 (0.7)
Cytotoxic non-MABs chemotherapeutic drugs	72 (51.1)
Azathioprine	14 (10.0)
Cyclosporine	3 (2.1)
Mycophenolate	5 (3.5)
Methotrexate	43 (30.5)
Anti-JAK	3 (2.1)
Another immunosuppressive drug	4 (2.8)

^1^ The overall percentage is higher than 100% because there are patients with more than one immunosuppressive treatment at the same time.

**Table 3:** Cumulative incidences and crude (unadjusted) risk ratios (RR) for non-response to vaccination against HBV.

Variable	Total (*n*)	Incidence of Non-Responders	cRR (95% CI)	Incidence of Persistent Non-Responders	cRR (95% CI)
**Exposure**					
IS treatment	141	36.9%	**2.33 (1.52–3.57)**	14.2%	**2.51 (1.17–5.41)**
No IS treatment	148	15.7%	Ref	5.4%	Ref
**Sociodemographic variables**					
Sex					
Men	173	25.2%	0.93 (0.54–1.60)	7.8%	0.71 (0.33–1.52)
Women	115	26.6%	Ref	11.0%	Ref
Age, *n* (%)					
<35 years old	47	10.6%	Ref	2.1%	Ref
35–45 years old	49	10.2%	0.96 (0.30–3.10)	8.2%	3.84 (0.44–33.09)
45–55 years old	80	26.3%	2.47 (0.99–6.11)	7.5%	3.53 (0.44–28.39)
55–65 years old	57	43.9%	**4.12 (1.71–9.93)**	15.8%	7.42 (0.98–56.48)
>65 years old	55	34.6%	**3.25 (1.31–8.03)**	14.6%	6.84 (0.89–52.68)
Smoking status, *n* (%)					
Non-smoker	86	18.8%	Ref	3.5%	Ref
Current smoker	49	34.7%	**1.84 (1.03–3.31)**	18.4%	**5.20 (1.48–18.32)**
Ex-smoker	44	47.7%	**2.54 (1.48–4.35)**	11.4%	3.22 (0.81–12.85)
**Main diagnosis ^1^**					
Arthritis/spondylitis	69	42.0%	**2.00 (1.37–2.92)**	15.9%	**2.05 (1.01–4.17)**
Digestive diseases	62	27.4%	1.07 (0.67–1.70)	3.2%	0.28 (0.07–1.15)
Dermatological diseases	47	14.9%	0.53 (0.26–1.07)	4.3%	0.39 (0.10–1.61)
CKD-dialysis	23	26.1%	1.00 (0.49–2.05)	13.0%	1.38 (0.45–4.23)
Systemic diseases	19	42.1%	1.69 (0.96–2.98)	26.3%	**3.08 (1.32–7.19)**
**Treatments (groups) ^1^**					
MABs	37	38.1%	**1.92 (1.31–2.81)**	13.4%	1.71 (0.85–3.44)
Anti-TNF	70	32.9%	1.38 (0.91–2.08)	12.9%	1.48 (0.70–3.11)
Anti-IL6 (tocilizumab)	6	66.7%	**2.65 (1.45–4.83)**	16.7%	1.74 (0.28–10.80)
Anti-CD20	5	60.0%	**2.36 (1.12–4.96)**	60.0%	**6.79 (3.03–15.23)**
Anti-IL17	7	14.3%	0.54 (0.09–3.37)	0.0%	-
Receiving ≥ 2 MABs	6	50.0%	1.95 (0.91–4.20)	16.7%	2.21 (0.51–9.52)
Cytotoxic non-MABs	30	41.7%	**1.99 (1.37–2.90)**	20.8%	**3.41 (1.73–6.74)**
Anti-JAK	3	100.0%	**2.93 (1.78–4.86)**	100.0%	**8.15 (4.53–14.67)**
Another IS	4	100.0%	**3.14 (2.03–4.84)**	25.0%	3.11 (0.78–12.42)
**Specific treatments ^1^**					
Adalimumab	58	31.0%	1.26 (0.81–1.95)	12.1%	1.37 (0.63–2.98)
Infliximab	9	66.7%	**2.62 (1.59–4.31)**	33.3%	**3.84 (1.53–9.66)**
Etanercept	5	20.0%	0.95 (0.24–3.86)	0.0%	-
Azathioprine	14	21.4%	0.89 (0.35–2.26)	14.3%	1.73 (0.53–5.67)
Methotrexate	42	48.8%	**2.21 (1.50–3.23)**	23.3%	**3.17 (1.60–6.30)**
Mycophenolate	5	60.0%	**2.29 (1.13–4.62)**	20.0%	2.58 (0.62–10.80)
**Baseline serological status ^1^**					
Negative (undetectable)	263	25.1%	Ref	8.4%	Ref
Unknown	23	39.1%	0.64 (0.37–1.09)	26.1%	0.31 (0.15–0.68)
**Vaccination received ^1^**					
Engerix	169	36.1%	**2.99 (1.78–5.05)**	13.6%	**3.02 (1.23–7.41)**
Vaxpro	14	0.0%	-	0.0%	-
Fendrix	27	29.6%	1.18 (0.65–2.14)	14.8%	1.72 (0.68–4.34)
Combined Vaxpro and Engerix	78	7.9%	0.26 (0.12–0.55)	1.3%	0.15 (0.03–0.76)

^1^ As these are dichotomous yes/no variables, reference values were omitted, as they are complementary to the values shown. For specific treatments, only those used in ≥5 patients in the sample were analyzed. Boldface type indicates 95% CIs that do not include the null value (1).

**Table 4:** Results of multivariable analysis for non-response to conventional vaccination.

Variable	cRR (95 CI%)	aRR1 (95 CI%)	aRR2 (95 CI%)	aRR3 (95 CI%)
Immunosuppression	2.33 (1.52–3.57)	**2.95 (1.59–5.48)**	**2.49 (1.25–4.96)**	**2.49 (1.26–4.96)**
Sex (male)	0.93 (0.54–1.60)	1.00 (0.56–1.79)	0.85 (0.47–1.56)	0.86 (0.47–1.58)
Age (quantitative)	1.06 (1.03–1.08)	**1.06 (1.03–1.08)**	**1.06 (1.03–1.08)**	**1.06 (1.03–1.09)**
Smoking status (current or ex-smokers)	2.98 (1.50–5.90)	**2.73 (1.27–5.84)**	**2.87 (1.33–6.21)**	**3.08 (1.41–6.74)**
Arthritis/spondylitis	2.73 (1.53–4.86)	**2.33 (1.24–4.36)**	**2.33 (1.24–4.36)**	1.49 (0.73–3.03)
Monoclonal antibodies	1.92 (1.31–2.81)	**2.31 (1.28–4.16)**	1.84 (0.94–3.57)	-
Tocilizumab (anti-IL-6)	2.65 (1.45–4.83)	3.73 (0.63–21.93)	2.92 (0.49–17.27)	-
Anti-CD20	2.36 (1.12–4.96)	3.67 (0.56–24.25)	3.87 (0.56–26.72)	-
Cytotoxic non-MABs	1.99 (1.37–2.90)	**2.60 (1.40–4.82)**	**2.24 (1.18–4.24)**	-
Anti-JAK	2.93 (1.78–4.86)	-	-	-
Methotrexate	2.21 (1.50–3.23)	**2.87 (1.40–5.89)**	2.17 (0.97–4.86)	-
Mycophenolate	2.29 (1.13–4.62)	2.42 (0.38–15.53)	3.23 (0.50–20.88)	-
Single Engerix regimen	2.99 (1.78–5.05)	**6.31 (3.07–13.00)**	**5.86 (2.83–12.13)**	**5.19 (2.46–10.92)**
Combined Vaxpro and Engerix regimen	0.26 (0.12–0.55)	**0.17 (0.01–0.14)**	**0.16 (0.06–0.41)**	**0.17 (0.07–0.43)**

aRR1: adjusted risk ratio for age and sex; aRR2: adjusted risk ratio for age, sex and baseline diagnosis; aRR3: adjusted risk ratio for age, sex, baseline diagnosis and immunosuppressive treatment. Results from the multivariable log-binomial (generalized linear model) regression models. The reference for each variable was its complementary variable (for example, “immunosuppression” shows the risk ratio of immunosuppressed patients compared to not immunosuppressed participants). Boldface type indicates 95% CIs that do not include the null value (1).

## Data Availability

Data will be available upon reasonable request from the corresponding author.

## Abbreviations

The following abbreviations are used in this manuscript:

antiHBc	Hepatitis B core antibodies
antiHBs	Hepatitis B surface antibodies
HBsAg	Hepatitis B surface antigen
HBV	Hepatitis B virus
JAK	Janus kinases
MAB	Monoclonal antibody
RR	Risk ratio

## Appendix A

**Table A1:** Monovalent hepatitis B vaccines available in Andalusia, Spain.

Vaccine (Company)	Dose	Indication
HBVAXPRO 10 (MSD^®^, Rahway, NJ, USA)	10 μg/mL of HBsAg, 1 mL	≥16 years old
ENGERIX-B (GSK^®^, London, UK)	20 μg/mL of HBsAg, 1 mL	≥16 years old
HBVAXPRO 40 (MSD^®^)	40 μg/mL of HBsAg, 1 mL	Adult patients on dialysis or pre-dialysis
FENDRIX (GSK^®^)	20 μg/mL of HBsAg, adjuvanted (AS04C 50 μg), 0.5 mL	Patients ≥ 15 years old on dialysis or pre-dialysis

**Table A2:** Vaccination received according to exposure (immunosuppression).

Vaccination Received	Total *n*	Exposed (Immunosuppressed), *n* (%)	Not Exposed (Not Immunosuppressed), *n* (%)	*p*-Value
Engerix	169	97 (57.4)	72 (42.6)	<0.001
Vaxpro	14	7 (50.0)	7 (50.0)	0.926
Fendrix	27	0 (0.0)	27 (100.0)	<0.001
Combined PC Vaxpro y Engerix	76	35 (46.1)	41 (54.0)	0.578
